# Cellular and molecular mechanisms of vitamin D in food allergy

**DOI:** 10.1111/jcmm.13607

**Published:** 2018-03-25

**Authors:** Ashlyn Poole, Yong Song, Helen Brown, Prue H. Hart, Guicheng (Brad) Zhang

**Affiliations:** ^1^ School of Public Health Curtin University Bentley WA Australia; ^2^ Centre for Genetic Origins of Health and Disease The University of Western Australia and Curtin University Crawley WA Australia; ^3^ Telethon Kids Institute The University of Western Australia Crawley WA Australia; ^4^ Curtin Health Innovation Research Institute Curtin University Bentley WA Australia

**Keywords:** epigenetics, food allergy, genetics, microbiome, vitamin D

## Abstract

Food allergies are becoming increasingly prevalent, especially in young children. Epidemiological evidence from the past decade suggests a role of vitamin D in food allergy pathogenesis. Links have been made between variations in sunlight exposure, latitude, birth season and vitamin D status with food allergy risk. Despite the heightened interest in vitamin D in food allergies, it remains unclear by which exact mechanism(s) it acts. An understanding of the roles vitamin D plays within the immune system at the cellular and genetic levels, as well as the interplay between the microbiome and vitamin D, will provide insight into the importance of the vitamin in food allergies. Here, we discuss the effect of vitamin D on immune cell maturation, differentiation and function; microbiome; genetic and epigenetic regulation (eg DNA methylation); and how these processes are implicated in food allergies.

## 1 INTRODUCTION

The incidence and prevalence of food allergies are increasing in Western societies, yet underlying mechanisms and risk factors are largely unknown.^1^, ^2^ Reported allergies to foods and hospital admissions for food‐related anaphylaxis have increased significantly since the early‐mid‐nineties in the United States, Europe and Australia, particularly in children.^3^, ^4^, ^5^, ^6^, ^7^ For example, in the United States, food allergy diagnoses rose 18% between 1997 and 2007 with increases in peanut and tree nut allergies in children most prominent, and hospitalizations and ambulatory care visits from food allergies have tripled since the mid‐nineties.^3^, ^4^ The United Kingdom has seen a 500% increase in hospitalizations from food allergy anaphylaxis since 1990, and cases in Australian children increased four fold from 1995 to 2006.^5^, ^6^

Food allergies reflect a lack of development of oral tolerance to food proteins, such as those found in milk, wheat, eggs, peanuts, tree nuts and soy.^8^ The most well‐known type is immunoglobulin E‐mediated (IgE‐mediated) food allergy. The mechanisms and developmental pathways of food allergies are complex and not well understood.^8^ Why some people develop food allergies is not clear, and the specific roles of several immune cells are not well defined.^9^ It is most likely that multiple pathways lead to a failure to develop tolerance to food antigens.

**Table 1:** Alleles and polymorphisms of genes with a potential role in food allergy

Gene	Function	Outcome	Potential role	Reference
*CYP27B1*	Activate vitamin D	Serum IgE	Genotype is associated with elevated IgE	39
*IL‐4*	Induces differentiation of Th0 cells into Th2 cells	Food sensitization	Vitamin D deficiency and CC/CT genotypes increase risk of food sensitization	17, 29
*MS4A2*	IgE receptor complex protein	Food sensitization	Vitamin D deficiency and GG genotype increases risk of food sensitization	17
*FCERIG*	IgE receptor complex protein	Food sensitization	Vitamin D deficiency and TT/TG genotypes increases risk of food sensitization	17
*CYP24A1*	Deactivate vitamin D	Food sensitization	Vitamin D deficiency and AA/AG genotypes increases risk of food sensitization	17
*GC*	Transports vitamin D in blood	Food allergy	Vitamin D insufficiency and GG genotype associated with food allergy	30
*IL‐13*	Induces IgE secretion and regulation of inflammation	Food allergy	CT genotype associated with elevated IgE and food allergy	65

Food allergies are multifactorial, and it has been proposed that the underlying mechanism is related to dietary composition, route and timing of allergen exposure, hygiene and microbiome, vitamin D, genetics and/or epigenetics.^10^ The past decade has seen an increase in the investigation into the apparent relationship between vitamin D and food allergies. Ecological studies have provided insight into the associations between sunlight, latitude and birth season with food allergy risk; all of which can impact vitamin D status.^11^, ^12^, ^13^, ^14^, ^15^ Furthermore, vitamin D status has been considered as a safe, cheap, modifiable risk factor for food allergy.^16^

In recent years, research has focussed on the mechanisms of this relationship by examining the role of vitamin D at both the gene expression and functional levels.^2^ This research has provided more evidence for the link between the vitamin and food allergies. Furthermore, modifications to the bioavailability and functioning of vitamin D via differences in the genes involved in vitamin D metabolism and function have been explored, not only in food allergies but in the general immune system.^17^, ^18^ This review will focus on the relationship between vitamin D and food allergy development, through a discussion of potential mechanisms of action at the cellular, genetic, epigenetic and microbial levels.

## 2 IGE‐MEDIATED FOOD ALLERGY

Food allergy describes an immune‐mediated reaction to a food. The term food allergy encompasses reactions classed as either IgE‐mediated or non‐IgE‐mediated, or both.^19^ Currently, IgE‐mediated food allergies are the best characterized and are the classically recognized type in society. IgE‐mediated food allergies can result in urticaria, angioedema, vomiting and/or anaphylaxis.^19^

Initial exposure to an allergen induces sensitization: the production of antigen‐specific IgE. Re‐exposure induces faster, larger reactions, as mediated by these IgE antibodies, characterizing food allergy. During re‐exposure in a previously sensitized individual, upon entering the body, the food allergen is taken up by dendritic cells which digest and present pieces of the antigen on their surface. Naïve T cells bind and differentiate into T helper cells, causing a cascade of immediate chemical and cellular responses. These responses include B cell differentiation, the production of antigen‐specific IgE and secretion of a range of cytokines and chemokines.^20^ The IgE‐triggered release of chemical mediators from granulocytes ultimately results in systemic symptoms that characterize an allergic reaction, including urticaria and anaphylaxis.^20^ These reactions are maintained by late‐phase chemokine and pro‐inflammatory cytokine secretion, and subsequent recruitment of additional leucocytes.^20^

## 3 VITAMIN D

Vitamin D encompasses both vitamin D_2_ (ergocalciferol) and vitamin D_3_ (cholecalciferol), as well as active derivatives. Endogenous production of vitamin D is the dominant source of the vitamin in humans, where 7‐dehydrocholesterol is converted to cholecalciferol, with UV‐B rays as the source of irradiation.^21^ The kidneys convert cholecalciferol into the active form, 1,25‐dihydroxyvitamin D, in a tightly controlled fashion.^21^ Additionally, there is increasing recognition into the localized activation of vitamin D in cells, including those of the immune system. The diet and supplements also supply vitamin D; however, these sources play little role in the vitamin D status of humans, particularly in regions that have no mandatory fortification programs, such as Australia and Europe.^21^, ^22^ Vitamin D insufficiency is reported to be highly prevalent even in populations with adequate sunlight, due to lifestyle changes, clothing and public health campaigns surrounding melanoma risk.^23^, ^24^

## 4 VITAMIN D IN FOOD ALLERGY EPIDEMIOLOGY

The vitamin D hypothesis of food allergies is supported by epidemiological evidence. In particular, associations between a range of environmental and biological factors with food allergy risk provide a link to vitamin D. Sunlight, latitude and birth season all impact vitamin D status and have each been shown to modify food allergy risk.^11^, ^12^, ^13^, ^14^, ^15^

Early epidemiological studies indicated some association between latitude and food allergy, and this could be explained by differences in endogenous production of vitamin D by exposure to UV rays. Latitude affects sunlight and solar radiation exposure, with the most southern and northern regions of the globe receiving fewer megajoules of sunlight per square metre.^12^ Populations of the United States and Australia in regions furthest from the equator have an increased risk of overall allergy, food allergy and food allergy markers, compared to those closest to the equator.^11^, ^12^ After controlling for population and region characteristics, one study reported prescriptions for epinephrine auto‐injectors were higher in the northernmost states of the United States, particularly those in the New England region (8‐12 prescriptions per 1000 people) when compared to southern regions (3 prescriptions per 1000 people).^11^ In an Australian study, similar prescriptions and admissions to hospital for anaphylaxis were higher in southern regions (Hobart, Tasmania) than northern regions (North Queensland).^12^ However, in this study, they were unable to differentiate between new and renewed prescriptions. Moreover, south‐eastern regions of Australia have greater prescriptions of hypoallergenic infant formula than other parts of the country, with rates as high as 14 406 per 100 000 people per year.^13^

Following observations of a low proportion of food allergies in children born in summer and spring months, it was hypothesized that birth season and food allergies were related. Researchers from the Australian HealthNuts study found the odds of food allergies in children born in summer were 55% lower than those born in other seasons.^14^ Similarly, another study found children born in winter and autumn, compared to those born in summer and spring, had higher rates of food allergies (57% vs. 43%), epinephrine auto‐injector prescriptions (54% vs. 46%) and prescriptions for hypoallergenic infant formula (54% vs. 46%).^15^ The authors reported significant associations of overall food allergy and ultraviolet radiation (UVR) intensity.^15^ Results from other studies have also suggested associations between vitamin D status at birth, as mediated by birth season, and the risk of developing food allergies later in life.^25^, ^26^ Atopic phenotypes appear to be developed in the crucial ante‐ and post‐natal periods, and as a result, the season of birth has an effect on overall immune development.^27^, ^28^

Several studies have identified an association between vitamin D insufficiency and deficiency and an increase in risk of developing food allergies. In one study, infants with low vitamin D (≤50 nmol/L) were 11 times more likely to have a peanut allergy, almost 4 times more likely to have an egg allergy, and more than 10 times more likely to have multiple food allergies when compared to infants with adequate vitamin D concentrations.^25^ Interestingly, these findings were only applicable to infants with parents born in Australia, rather than those who had migrated to Australia.^25^ Both Liu et al^29^ and Koplin et al^30^ observed an association between prolonged vitamin D insufficiency and food sensitization, but found no associations between vitamin D levels and allergy when analysed at single points in time. An observational study by Jones et al^26^ demonstrated an association between high vitamin D status at birth and a decrease in markers of allergic reactivity to egg protein while the child was below 6 months of age. These effects, however, were not replicated when testing was performed in infants above 6 months of age, or for milk protein allergy markers.^26^ According to results from the National Health and Nutrition Examination Survey in the United States, there were no associations between egg and milk allergies with vitamin D status, but those with vitamin D deficiency were 2.39 times more likely to have a peanut allergy.^31^

In contrast, some European studies found vitamin D supplementation and a higher vitamin D status increased risk of allergy. A cohort study conducted in Finland investigated the association between vitamin D supplementation in infancy (beginning 1966) and allergy outcome at 31 years of age.^32^ Those who received regular vitamin D supplementation in infancy had a greater risk of overall allergy (OR 1.46); however, allergies to food proteins were not explored.^32^ Recently, Junge et al^33^ showed that German infants with vitamin D in the highest quartile at birth had an increased risk of food allergies at 3 years of age (OR 1.86). Similarly, another German study found maternal and cord blood vitamin D levels were positively associated with risk of food allergy within the first 2 years of age.^34^ However, this is not consistently observed and, furthermore, other studies have found no significant association between vitamin D status and food allergies.^17^, ^35^

Despite more evidential weight in the risk of food allergies in those with vitamin D insufficiency than oversupply, a U‐shaped curve has been proposed. This proposal describes a U‐shaped relationship between vitamin D status and food allergy predisposition, where too little or too much vitamin D provides the greatest risk.^36^, ^37^, ^38^ In a biochemical plausibility context, the U‐shaped curve for allergies is supported by the discovery of a nonlinear relationship of vitamin D concentrations with IgE levels, of which are used as a marker of allergic status.^39^, ^40^ However, a recent review determined that there was not enough longitudinal or controlled data on allergies and vitamin D to confirm the shape of the relationship, nor was there sufficient evidence to suggest the U‐shape of vitamin D in relation to the risk of any disease investigated, including food allergies.^38^

## 5 VITAMIN D MODULATION AT THE CELLULAR LEVEL

The active form of vitamin D (1,25 dihydroxy vitamin D) has direct and indirect effects on the function of immune cells. Alterations in vitamin D status affect development and function of key immune cells, including the T cells, dendritic cells and regulatory T cells (Tregs).^41^, ^42^, ^43^ This affects allergic responses through modulation of immune mediators such as IgE and pro‐ and anti‐inflammatory cytokines.

Tregs are important immune regulators, having the ability to suppress inflammatory responses and promote allergen tolerance through a range of actions including the secretion of anti‐inflammatory cytokines, such as IL‐10.^8^, ^44^ An absence of Tregs is a key issue in those with food allergies, with low Tregs and IL‐10 exacerbating hypersensitivity.^8^, ^45^ The relationship between vitamin D levels and Tregs is unclear. In patients with multiple sclerosis, Smolders et al^46^ reported a positive association between vitamin D and Treg number and function. However, in later research, the same team found no association.^47^ In food allergy research, cord blood vitamin D levels were negatively correlated with Treg numbers, although in this case the trend was weak.^34^ More recently, a mouse study showed vitamin D‐deficient females had pups that were at a greater risk of food sensitization and had suppressed Treg cells, compared to the pups that were from females fed a balanced diet.^48^ The relationship between vitamin D levels and Tregs is better described when considering the involvement of dendritic cells (DCs). Vitamin D inhibits DC maturation and differentiation.^43^, ^49^ Stimulation of DCs by vitamin D promotes the development of Tregs.^42^ Additionally, Tregs and IL‐10 can be modulated via Toll‐like receptor (TLR) pathways.^16^, ^50^

The TLRs have definitive roles in innate immunity and have been implicated in allergic disease.^51^ TLR expression can be modulated by vitamin D, and ligands of TLRs are related to both vitamin D metabolism and innate immune responses.^52^ Ex vivo, expression of *TLR7* was positively associated with serum vitamin D levels, and TLRs 1, 2, 3, and 6 were negatively correlated.^16^ Expression of *TLR2* and *TLR4* is down‐regulated by vitamin D in several studies.^16^, ^53^, ^54^ Conversely, in neutrophils cultured with *Mycobacterium tuberculosis,* expression of *TLR2* and *TLR9* is significantly up‐regulated by vitamin D.^55^ Stimulation of TLRs can up‐regulate expression of the VDR and the protein that activates vitamin D, 25‐hydroxyvitamin D‐1 alpha‐hydroxylase (CYP27B1).^56^ Additionally, the binding of ligands to TLR2 and TLR4 induces cytokine production.^52^, ^57^

Markers of sensitization and allergy, namely IgE and cytokines, have been explored in relation to vitamin D. Overproduction of IL‐4 and subsequent IgE production is a major characteristic of allergic status.^50^ An examination of data from the National Health and Nutrition Examination Survey revealed that serum vitamin D levels are inversely proportional to total IgE levels.^37^ In B cells, vitamin D inhibits IgE production and promotes anti‐inflammatory IL‐10 through local activation and binding to the VDR.^50^ After adjustment for factors such as sex, lifestyle, geographical location and month of blood draw, a cross‐sectional study found IgE concentrations were 29% higher in participants with vitamin D deficiency (25(OH)D < 25 nmol/L) than the group with sufficient levels of vitamin D.^39^ Furthermore, IgE levels were 56% higher in the group of participants with the highest vitamin D concentrations (25(OH)D > 135 nmol/L), indicating a nonlinear relationship and threshold effect of vitamin D and IgE.^39^

Down‐regulation of TLR4‐mediated IL‐1B, IL‐6, IL‐10, IFNγ and TNFα production was associated with higher serum vitamin D levels and summer months in an ex vivo study by Khoo et al.^57^ However, they found little seasonal effect on TLR2 responses.^57^ A review of cell studies reported that production of TNFα is induced, and IFNγ is inhibited, by vitamin D, and vitamin D can interfere with a range of immune cell signalling processes, including phosphorylation and translocation.^58^ In contrast, a randomized placebo‐controlled trial demonstrated that vitamin D supplementation for 6 months had no effect on the expression of IFNγ or other cytokines in vitamin D‐deficient women.^59^

## 6 VITAMIN D MODULATION AT THE GENETIC LEVEL

**Figure 1:**
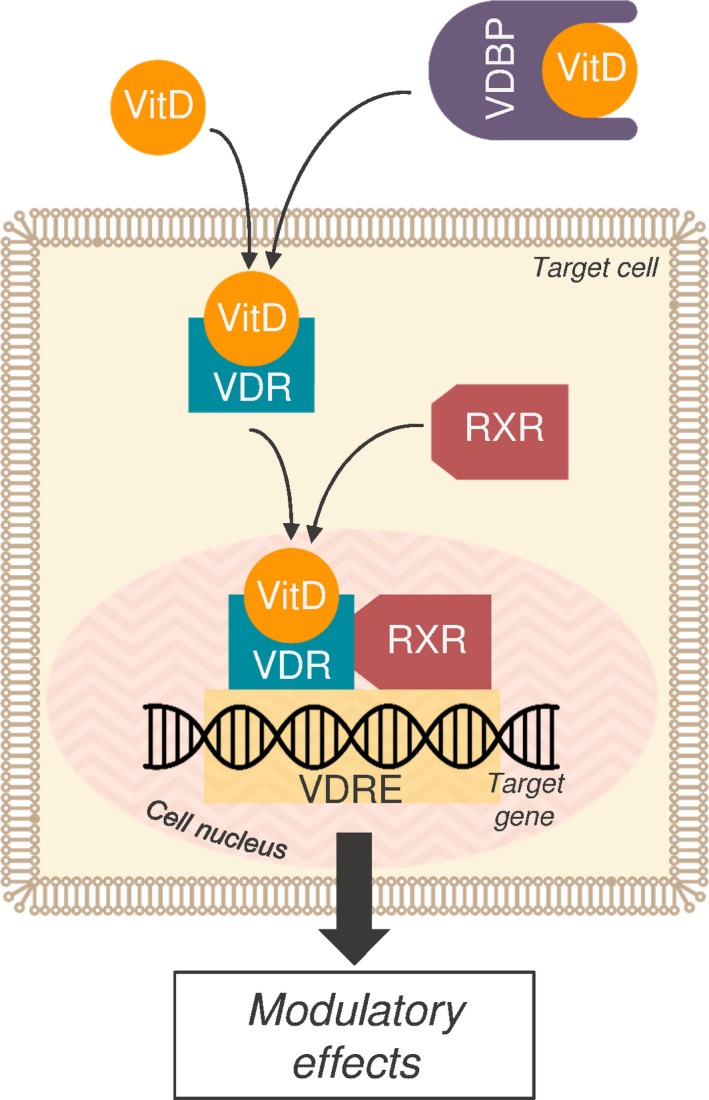
Vitamin D action on target genes. Transported throughout the body unbound or bound to vitamin D‐binding protein (VDBP), vitamin D (1,25(OH)2D) enters the target cell and binds to the vitamin D receptor at the nuclear membrane. In the nucleus, this forms a heterodimer with retinoid X receptor and binds to the vitamin D response element in the promoter region of target genes. Gene expression is altered and modulatory effects take place. RXR, retinoic X receptor; VDBP, vitamin D‐binding protein; VDR, vitamin D receptor; VDRE, vitamin D response element; VitD, vitamin D

Vitamin D predominantly modulates immune activity through its action on responsive genes. The downstream target genes typically harbour a vitamin D response element in the promoter region (Figure 1). After active vitamin D binds to the VDR, the heterodimer of VDR complex and retinoid X receptor binds to the vitamin D response element and induces expression of these target genes.^60^ Most cells of the immune system express VDR, including T cells and antigen‐presenting cells, and possess the ability to convert vitamin D into its active form locally, leading to an increased interest in the role of the vitamin in immune modulation.^58^, ^60^, ^61^

Genetic studies have suggested that multitudes of genes could be involved in the development of allergic disease, including genes associated with vitamin D metabolism, and skin and gut barrier integrity.^62^ Specifically, the vitamin D response element has been identified in several genes directly relevant to food allergy pathogenesis, including those encoding for cytokines *TNF‐*α, *IFN‐*γ, *IL‐10*, and the antigen receptor proteins *HLA‐DRB1* and *HLA‐DQA1*.^44^

Alleles in *CYP27B1* have been shown to modify vitamin D and IgE responses in an observational study, with the A allele associated with elevated IgE and 25(OH)D concentrations.^39^ Later, Liu et al^17^ attributed variance in food sensitization risk to polymorphisms in genes relating to vitamin D metabolism and allergic response. Vitamin D deficiency was reported to be associated with alterations in IgE receptors, including *FCERIG* and *MS4A2* and CC/CT genotypes of the *IL‐4* gene, resulting in an increased risk of developing food sensitization in those with vitamin D deficiency.^17^ Subsequently, the C allele of the *IL‐4* gene was specifically found to increase the risk of food sensitization from low vitamin D.^29^

Alleles of genes encoding proteins involved in vitamin D metabolism and functioning, such as *GC*,* DHCR7*,* CYP2R1* and *CYP24A1,* have been associated with a risk of vitamin D insufficiency and may be further implicated in food sensitization (Table 1).^63^ Variations in the gene that encodes for vitamin D‐binding protein (VDBP), *GC*, could implicate the role of vitamin D in the immune system. Research on ovalbumin‐sensitized mouse models reported increased expression of *GC* when compared to sham mice, and treatment with anti‐VDBP antibodies reduced ovalbumin‐induced airway hyper‐responsiveness in a dose‐dependent manner.^64^ The anti‐VDBP antibody also attenuated the effects of VDBP on a number of inflammatory cells including eosinophils, neutrophils and lymphocytes.^64^ A recent study hypothesized that single nucleotide polymorphisms of two main alleles of the *GC* gene decreased levels of VDBP which would subsequently increase the bioavailability of circulating vitamin D, thereby compensating for the effects of decreasing serum vitamin D on food allergy risk.^30^ Results from the study indicate the GG genotype modifies the association between low vitamin D status and food allergy.^30^ Additionally, the relationship between maternal antenatal vitamin D supplementation was more pronounced in infants with the GT/TT genotypes.^30^

Other genetic modifications have also been explored, with single nucleotide polymorphisms at *IL‐13*,* HLA‐DR*,* HLA–DQ*,* MS4A2*,* FCERIG* and *CYP2A1* associated with food sensitization or food allergy, and vitamin D.^17^, ^65^, ^66^ These genetic factors may be used to predict outcomes of vitamin D deficiency or supplementation on allergy risk in future trials.

## 7 VITAMIN D MODULATION AT THE EPIGENETIC LEVEL

Modulation of food allergy by vitamin D is primarily achieved through its action as a transcription factor; however, not all genes regulated by vitamin D contain a vitamin D response element (VDRE), indicating some additional pathways of transcriptional modulation.^33^, ^67^ Epigenetic regulation of gene expression may be a key explanation for the association between environmental factors and food allergy development. Risk factors such as breastfeeding duration, exposure to animals and early life infection have been implicated in the development of food allergies for susceptible individuals and epigenetic modifications may be the mechanism for these associations.^62^

DNA methylation is the most commonly known type of long‐term epigenetic modification, with transcriptional activity being directly affected by level of methylation.^68^ Methylation and demethylation of DNA have been implicated in food allergy through several mechanisms yet the role of vitamin D has been seldom explored.^69^ Junge et al^33^ highlighted the significance of the relationship between vitamin D levels and a gene involved in allergic inflammation, thymic stromal lymphopoietin (*TSLP*). It was reported that methylation of an enhancer region near the *TSLP* promoter region correlated with vitamin D in children with high cord blood vitamin D status, irrespective of genetic variation.^70^ *TSLP* has been reported to promote allergic sensitization through its effects on dendritic cells and basophils, and production of IL‐4.^71^

Genes involved in vitamin D metabolism and function, such as *VDR, CYP2R1* and *CYP24A1,* are particularly susceptible to DNA methylation due to the presence of large cytosine‐guanine islands in the genes.^72^ A genome‐wide methylation study found the genes *DHCR7, CYP2R1* and *CYP24A1* in leucocytes were differentially methylated between participants with vitamin D deficiency compared to those with adequate vitamin D status.^73^ Modulation of vitamin D activity by epigenetic DNA methylation may have indirect effects on development of food allergies.

Vitamin D itself may additionally influence DNA methylation. In an assessment of epigenetic outcome, maternal vitamin D deficiency led to alterations in DNA methylation covering two generations of Collaborative Cross mouse pups.^74^ Conversely, a genome‐scale assessment of mononuclear blood cells found no substantial change in DNA methylation, although vitamin D‐dependent genes were indeed up‐regulated.^75^ Thus, it is not yet obvious whether there is a direct effect vitamin D on DNA methylation.

## 8 VITAMIN D MODULATION VIA MICROBIAL PATHWAYS

The role of the microbiome in regulation of immunity is of great interest in current research. The hygiene hypothesis of food allergies described a link between cleanliness and risk of food allergy.^10^ As more information was uncovered, the intestinal microbiome became a key part of this hypothesis.^76^ There have been emerging insights into how vitamin D interacts with the microbiota of the host.

Vitamin D has a well‐established role in the regulation of antimicrobial peptides, such as cathelicidin, produced in both the gut and the skin.^77^ In the intestinal lining, this modulation of antimicrobial peptide synthesis by vitamin D affects the homeostasis of the gut barrier. In an *in vitro* study, cathelicidin maintained the integrity of the intestinal lining both directly and indirectly.^78^ Intestinal epithelial cell migration and the expression of protective mucins were enhanced by cathelicidin.^78^ In a mouse model, vitamin D deficiency compromised the mucosal barrier, leading to increased susceptibility to mucosal damage.^79^ Therefore, attenuation of antimicrobial capacity and injury to the protective barriers of both the gut and the skin raise a plausible mechanism of vitamin D underlying the development of food allergy.

Disruption to the gut barrier also leads to a dysbiosis of microbiota.^76^ Intestinal dysbiosis increases susceptibility to pathogens and toxins and triggers inflammatory responses, and it is proposed that this cascade can lead to food allergies.^80^ Studies in mice have shown a link between vitamin D deficiency, or VDR suppression, and alterations to gut microbe composition.^81^ *Clostridium* and *Bacteroides* were depleted in faeces in one study of VDR deficient mice, whereas *Lactobacillus* was enriched.^82^ Through investigation of vitamin D signalling on the microflora of mice with insulin resistance, it was shown that vitamin D is necessary in the maintenance of the interface between the intestinal epithelia and gut microbiota.^83^ In the VDR knockout mice, composition of the gastrointestinal tract was significantly modified, with increased abundance of pathogenic bacteria and suppressed symbiotic bacteria, and changes in expression of defensin peptides, mucosal genes and tight junction genes.^83^ Vitamin D deficiency at birth resulted in long‐term alterations to colonic bacteria in mice, resulting in a susceptibility to the inflammatory state.^84^

The effect of vitamin D on microbial composition in humans is less obvious as studies are limited. A pilot trial on the effects of vitamin D on gut microbiome in healthy adults demonstrated significant alterations to microbial composition in the upper gastrointestinal tract after 8 weeks of vitamin D supplementation, but less so in other areas.^85^ Relative abundance of Gammaproteobacteria decreased significantly, and there was an increase in microbial diversity.^85^ Most recently, vitamin D levels in utero were associated with variations in bacteria from the *Firmicutes* phylum by 6 months of age; however, supplementation with vitamin D after birth had no significant effect.^86^

Changes to microbial composition can affect immunity, with intestinal microbiota inducing Treg and Th1 cell differentiation, and promoting Th1 cell responses.^76^ Tregs, and the anti‐inflammatory cytokines they release, are critical in the suppression of effector T‐cell responses that lead to allergic disease.^76^ Through dysbiosis, the microbiome can play a significant role in the development of allergic disease.

## 9 SUMMARY

Increased data showing a link between vitamin D insufficiency and food allergies have prompted investigations into the underlying mechanism(s). Epidemiology, animal, human and genetic studies appear to support a role of vitamin D in the development of food allergies. Populations with lower levels of vitamin D, including those living furthest from the equator and those in early infancy, are more likely to develop allergies to foods. There appears to be a protective role of vitamin D in food allergy risk.

Variants of certain genes involved in vitamin D metabolism alter vitamin D status and responsiveness to changes in vitamin D by supplementation and appear to make some individuals more susceptible to food sensitization. Additionally, there are many genes potentially modulated by vitamin D that play a direct or indirect role in food allergy pathogenesis. Many of the genes containing VDRE are located within the immune system. Genetic predisposition to food sensitization and allergy may be modulated by epigenetic interactions, and early life vitamin D status may affect development of immunity via epigenetic mechanisms. Furthermore, the role of the microbiome is of increased interest.

It is likely there is no single mechanism for the apparent relationship between vitamin D and food allergies, but many. Vitamin D status in early life appears to have a profound effect on the longer term immune health. The effect of vitamin D on immune cell signalling and function is important. Novel pathways, such as cathelicidin, must be explored further. Understanding of these mechanisms is essential for the investigation into the potential therapeutic role of vitamin D for food allergy.

## CONFLICTS OF INTEREST

The authors confirm that there are no conflicts of interest.
